# GPR37 Signaling Modulates Migration of Olfactory Ensheathing Cells and Gonadotropin Releasing Hormone Cells in Mice

**DOI:** 10.3389/fncel.2019.00200

**Published:** 2019-05-09

**Authors:** Hassan Saadi, Yufei Shan, Daniela Marazziti, Susan Wray

**Affiliations:** ^1^Cellular and Developmental Neurobiology Section, National Institute of Neurological Disorders and Stroke, National Institutes of Health, Bethesda, MD, United States; ^2^Consiglio Nazionale delle Ricerche, Emma-Infrafrontier-Impc, Istituto di Biologia Cellulare e Neurobiologia, Monterotondo Scalo, Rome, Italy

**Keywords:** GPR37, GnRH, olfactory ensheathing cells, migration, olfactory system

## Abstract

Gonadotropin releasing hormone (GnRH) neurons, part of the hypothalamic-pituitary-gonadal axis, regulate reproduction. Prenatally, GnRH neurons migrate into the brain from the nasal placode along terminal nerve fibers, intermixed with olfactory sensory axons and olfactory ensheathing cells (OECs). An expression analysis from embryonic GnRH neurons identified the G protein-coupled receptor 37 (GPR37 or PAEL-r). GPR37 has been linked to (1) juvenile Parkinson’s disease in humans, (2) oligodendrocyte differentiation, and (3) Wnt/β-catenin signaling during neurogenesis. In this study, the role of GPR37 was investigated in the developing GnRH/olfactory system. PCR and immunocytochemistry confirmed expression of GPR37 in migrating GnRH neurons as well as in OECs. Inhibition of GPR37 signaling in nasal explants attenuated GnRH neuronal migration and OEC movement. Examination of GPR37 deficient mice revealed a decrease in the olfactory bulb nerve layer and attenuated/delayed maturation and migration of GnRH neurons into the brain. These data demonstrate a developmental role for GPR37 signaling in neural migration.

**Significance Statement:**

Reproduction is controlled by gonadotrophin releasing hormone (GnRH) neurons located in the central nervous system. Embryonically, GnRH neurons originate in the nasal/olfactory placode and migrate into the brain on axonal tracks from cells in the vomeronasal organ, intermixed with olfactory sensory axons and olfactory ensheathing cells (OECs). An expression analysis from embryonic GnRH neurons identified the G protein-coupled receptor 37. Here we show that inhibition of GPR37 signaling in nasal explants and mutant mice attenuated GnRH neuronal migration. Signaling via GPR37 also perturbed OEC movement, resulting in a decrease in the olfactory bulb nerve layer *in vivo*. Together, these results identify a new role for GPR37 signaling during development – modulating cell migration.

## Introduction

GnRH neurons form a functional circuit as part of the hypothalamic-pituitary-gonadal axis and control reproduction. Embryonically, GnRH neurons originate in the nasal/olfactory placode. To access the forebrain, GnRH cells migrate across the nasal region and through the cribriform plate on axonal tracks from cells in the vomeronasal organ (VNO) (Wray, 2010). Upon entering the CNS, vomeronasal/olfactory axons infiltrate the olfactory bulbs, whereas GnRH neurons migrate caudally on a subset of non-sensory olfactory axons toward the hypothalamus (Forni et al., 2011). Disruption of the GnRH system (i.e., improper migration during early development) causes reproductive disorders and is often associated with anosmia due to disruption of the development of the olfactory system. Genetic studies of human patients with hypogonadotropic hypogonadism and anosmia, Kallman’s Syndrome, have identified a variety of molecules that influence GnRH neuronal migration and/or olfactory development (Wray, 2010; Topaloglu, 2017; Bouilly et al., 2018). To uncover novel regulators of GnRH neuronal development, a microarray screen was performed (Kramer and Wray, 2000; Dairaghi et al., 2018), and identified G protein-coupled receptor 37 (GPR37). Data from the Allen Developing Mouse Brain Atlas (2008) (Sunkin et al., 2013) and the Gene Expression Nervous System Atlas (GENSAT) project (Gong et al., 2003) confirmed developmental expression of GPR37 transcript and protein, respectively, in nasal regions that corresponded to the migratory route of GnRH neurons in mice.

GPR37, a parkin-associated endothelial-like receptor (PAELR), has been linked to juvenile Parkinson’s disease in humans (Takahashi and Imai, 2003; Imai et al., 2007) and Parkinson disease models in mice (e.g., MPTP treated), where its absence protected against dopaminergic cell death (Marazziti et al., 2004; Mandillo et al., 2013). Prosaposin (PSAP, also known as sulfated glycoprotein-1), and its active fragment Prosaptide (TX14A), mediate extracellular-signal-regulated-kinase 1/2 (ERK) signaling and cellular protection through stimulation of GPR37 (Meyer et al., 2013, 2014). Postnatally, GPR37 is expressed in neurons in “Parkinson’s disease-associated” nuclei including the caudate, putamen, and substantia nigra (Donohue et al., 1998; Imai et al., 2007) and modulates dopaminergic neurotransmission, either by changing dopamine levels (Marazziti et al., 2004; Imai et al., 2007), reducing postsynaptic sensitivity of striatal dopamine receptors (Marazziti et al., 2007) and/or inhibiting adenosine A_2A_ receptor cell surface expression and function in the striatum by forming GPR37/A_2A_R heteromers (Morato et al., 2017). GRP37 is also linked to developmental events. GPR37 is expressed in oligodendrocytes (Smith et al., 2017), and a GPR37-mediated control of oligodendrocyte differentiation has been described (Yang et al., 2016). GPR37 is also expressed in neural progenitor cells, where it acts as an endoplasmic reticulum chaperone for Low Density Lipoprotein Receptor-Related Protein 6 (LRP6), and thus is important for Wnt/β-catenin signaling during neurogenesis (Berger et al., 2017). In addition, GPR37 has been implicated in autism spectrum disorder (Fujita-Jimbo et al., 2012), a neurodevelopmental disorder for which dysfunction of several genes associated with axonal guidance signaling pathway has been suggested (Sbacchi et al., 2010).

This study evaluated the role of GPR37 signaling in the developing olfactory-GnRH system in mouse using both *in vitro* and *in vivo* models with wild type and GRP37 knockout mice. The data show that (1) GPR37 is expressed in both GnRH cells and OECs, (2) perturbation of GPR37, genetically or pharmacologically, attenuated OEC and GnRH neuronal migration, and (3) the olfactory nerve layer decreased in GPR37 KO mice into adulthood. These results indicate a new role for GPR37 signaling during development – modulating cell migration.

## Materials and Methods

### Animals/Tissue Collection

All animal procedures were in accordance with the National Institutes of Health, National Institute of Neurological Stroke and Disorders guidelines. Mouse lines used included NIH Swiss and GPR^tm2Gtva^ (GPR37 KO, accession ID no. MGI 3027995); the latter kindly provided by Marazziti et al. (2004). Mice were euthanized in a CO_2_ chamber followed by cervical dislocation, and then removal of embryos, testis, and/or brains. Embryos (E11.5–E18.5) and adult brains were removed and either immediately frozen and stored at -80°C or fixed (10% formalin, 4°C overnight), cryoprotected (30% sucrose/PBS, 4°C overnight), embedded (Tissue-Plus O.C.T.), and stored (-80°C). Serial sections (10–20 μm thickness) were cut on a Leica CM 3050S cryostat (Leica Biosystems) and maintained at -80°C until processing for immunocytochemistry (ICC). PCR analysis for verifying genotype was performed using three primers: P1-forward primer (5′-CATTGACCCAAGAATCCTACG-3′), P2-forward primer (5′-CTGTTCCATAGTTAACCT-AGC-3′), and P3-reverse primer (5′-CAGGCT-AGGAGCAATGGAG-3′) to amplify a 443 bp fragment specific to WT and a 245 bp fragment specific to the disrupted GPR37 allele (Marazziti et al., 2004). Note: In our animal facility, GPR37^-/-^ and GPR37^+/-^ mice did not produce many successful time matings, i.e., left together for 12 h. In contrast, these mice were able to routinely mate when left together for at least 3 days.

### Primary Nasal Explants

Explants were cultured as previously described (Fueshko and Wray, 1994; Klenke and Taylor-Burds, 2012). Briefly, embryos were removed from time-pregnant NIH-Swiss at E11.5, the nasal region containing the olfactory pits was dissected and placed in Gey’s balanced salt solution (Life Technologies, Inc.) supplemented with glucose (Sigma Chemical Co.). The sex of the embryos was not determined. Each embryo generated one nasal explant. Nasal explants were adhered to coverslips by a chicken plasma (Cocalico Biologicals)/Thrombin (Sigma) clot and maintained at 37°C in serum-free media (SFM) in a humidified atmosphere with 5% CO_2_. The medium was changed after 3 days and supplemented with one dose of fluorodeoxyuridine (4–10 μM; Sigma) to inhibit proliferation of dividing olfactory neurons and non-neuronal tissue (e.g., fibroblasts).

### Primary Cultures of OECs

Cultures of OECs were generated from olfactory bulbs of up to 1 week old neonatal mice. Cells were purified by the differential cell adhesion method (Nash et al., 2001) and cultured for up to 2 weeks. Briefly, olfactory bulbs were collected and degraded in an enzyme mix [hyaluronidase (Sigma), dispase I (Sigma), collagenase type 4 (Worthington), DNAse (Worthington)] (Au and Roskams, 2002) for 35 min (37°C) with constant agitation. Cells were strained through a 40 μm cell strainer to remove non-dissociated tissue pieces and then washed with media [Dulbecco’s minimum essential media (Gibco) and Ham’s F-12 (Gibco), at a 1:1 mixture with 10% fetal bovine serum (Gibco), 1% antibiotic mixture (PSN, Gibco)] to remove enzyme residues. Re-suspended cell solution (4 × 10^6^ viable cells/flask) was seeded into uncoated T75 flasks for 18 h to remove fast attaching fibroblasts. The supernatant of the first flask was seeded into another uncoated flask for up to 36 h to allow attachment of astrocytes. The final supernatant was seeded onto poly-L-lysine (Sigma)-coated flasks to grow primary OECs. Media was changed every 2–3 days.

### PCR on OECs, GnRH Cells, Nasal Explants, and Embryonic cDNAs

OEC were trypsinized and processed for RNA extraction (RNAqueous kit; Ambion). Following extraction, cDNA was created using Moloney murine leukemia virus reverse transcriptase (SuperscriptTM III; Invitrogen) according to manufacturer’s protocol. The purity of OECs was determined to be more than 90% based on p75 and S100B immunostaining. cDNAs from single GnRH cells (Kramer et al., 2000) were analyzed using microarrays (Dairaghi et al., 2018). Chip data sets were screened for novel modulators of neuronal migration based on high expression. GPR37 and its ligand, Prosaposin (PSAP), were identified and chosen for further investigation. Transcript analysis via PCR was performed on cDNAs generated from single GnRH cells maintained in explants, whole explants and embryos at different stages (Sharifi et al., 2002; Klenke et al., 2014). Primers used are listed in Table 1.

**Table 1:** Primer sets.

Gene	Forward	Reverse
GnRH (220 bp)	5′-CTGATGGCCGGCATTCTACTGC-3′	5′-CCAGAGCTCCTCGCAGATCCC-3′
GPR37 (182 bp)	5′-GGCCAACAGTCTCAGATCAT-3′	5′-GTACTTCCAATCACAACTCAAACAC-3′
PSAP (219 bp)	5′-TCCATTCTGCTTTCCTGTCTTC-3′	5′-GTGAACAGGCAGGTCAACAA-3′
S100 (109 bp)	5-AGAGGGTGACAAGCACAAGC-3′	5-ACTTTGTCCACCAACTTCCTGC-3′
Mbp (282 bp)	5′-GAGACCCTCACAGCGATCCAAG-3′	5′-GGAGGTGGTGTTCGAGGTGTC-3′
P75NGFR1 (264 bp)	5′-TGC AAT TAG TAG AAG GAC CCC ACC-3′	5′-TAC ACA GGA TAG CAA AGG GGA-3′


### Immunocytochemistry (ICC)

Primary antibodies used were: rabbit polyclonal (Rb) anti-GnRH (SW-1, 1:5000–15,000) (Wray et al., 1988), mouse monoclonal anti-GnRH (FID3C5, 1:4000 gift from Dr. Karande), Rb anti-Peripherin (peripheral intermediate filament marker; 1:2000; Chemicon), Rb anti-GPR37 (GPR37-3; 1:1000; MAb Technologies), Rb anti-PSAP (1:300, Proteintech), goat anti-Sox10 (1:150, SantaCruz), and Rb anti-S100ß (1:4000, Dako), goat anti-OMP (1:2000, gift from Dr. Margolis), and mouse monoclonal anti-reelin (1:100, G10, Millipore). Chicken anti-GnRH (1:100, Aves), chicken anti-peripherin (1:1000, Aves) and Guinea pig anti-S100B (1:500, Synaptic systems) were used for double labeling when staining for GnRH (or peripherin) with GPR37 (or PSAP). Explants and embryos were stained as described previously (Fueshko and Wray, 1994; Forni et al., 2011). In brief, explants were fixed (10% formalin, 1 h, room temperature). Slides of previously sectioned fixed and fresh frozen tissue were, similarly, fixed at room temperature, 10 min or 1 h, respectively. After PBS washes, the tissues were blocked (10% normal horse serum/0.3% Triton X-100, 1 h) and washed in PBS. Prior to blocking, fixed tissue sections were treated to block endogenous peroxidase activity using hydrogen peroxide suppression if using a chromogen reaction (15 ml methanol, 35 ml PBS, 0.5 ml 30% H_2_O_2_, 20 min), and subjected to antigen retrieval, as previously described (Shi et al., 1993). The tissues were then incubated in primary antiserum for 1–2 days at 4°C. After incubation, standard protocols for chromogen staining were used: biotinylated secondary antibodies were donkey anti-species specific-bt (1:500, Jackson ImmunoResearch Laboratories, Inc.), visualized with ABC-peroxidase (Vector laboratories)/chromogen methods (Fueshko and Wray, 1994). For fluorescent staining, secondary antibodies were directly conjugated anti-species-specific Alexa Flour 488 and 555 (1 h, 1:1000, Invitrogen). Controls were run with experimental tissues and consisted of tissue incubated in PBS/BSA instead of primary antibody for single labeling and tissue incubated in PBS/BSA instead of 2nd primary for double labeling to ensure specificity. Images were taken using iVision software (BioVision) either on a Nikon Eclipse E800 with Retiga SRV camera or a spinning disk confocal system CSU10 (Yokogawa) mounted on an Eclipse TE200 microscope (Nikon) using an EMCCD ImageM digital camera (Hamamatsu). Images for Supplementary Figure 2 are surface renderings of images shown in Figure 3 (E11.5), processed using Imaris 9.1 (Bitplane Zurich, Switzerland). Thresholding was done by background subtraction using local contrast. In red (peripherin), the threshold value was set at 600 and smoothing was done with a surface grain size set at 0.5 μm. In green (GPR37), the threshold value was set at 180 and smoothing was done with a surface grain size set at 1 μm. In both channels, volumes had to at least 200 voxels to be displayed.

### Verification of GPR37 Antibody

As noted above, time matings with the GPR37 mouse line were rarely successful, and thus few explants were obtained from GPR37^-/-^ embryos. However, 2 explants from different litters were used with explants from WT littermates for verification of antibody staining *in vitro*. No staining was detected in tissue from GPR37^-/-^ embryos, while robust labeling of cells was detected in tissue from WT littermates (Supplementary Figure 1). In addition, staining of adult olfactory bulbs from WT and KO mice was also performed, with a no primary control (Supplementary Figure 1). Staining in the olfactory nerve layer was only detected in tissue from WT adults exposed to the GPR37 antibody.

### Functional Assays

Two different assays were used in explants generated from NIH Swiss embryos: (1) chronic treatment of explants followed by *post hoc* analysis of total change in the location of GnRH neurons, OECs, and olfactory axons, and (2) live imaging of explants to determine acute changes in GnRH neuronal migration rates. Both assays employed Prosaptide TX14(A) and Macitentan. Tx14A is a GPR37 agonist (Meyer et al., 2013). No specific GPR37 antagonist have been identified. However, GPR37 is ∼40% homolgous with the endothelin-B receptor (Marazziti et al., 1997). Thus, macitentan (Mac) a dual endothelin receptor antagonist with a high affinity (Howell et al., 2014) was used.

#### Chronic Treatment

Nasal explants were treated 1 and 3 div with Prosaptide TX14(A) (TX14A, 1 μM, a GPR37 agonist; Tocris), Macitentan (Mac, 1 μM, Endothelial A/B receptor Antagonist, APExBIO), or vehicle medium, fixed at 7 div, and processed by immunocytochemistry for GnRH and/or peripherin and OECs to assess overall GnRH neuronal migration, olfactory sensory axon outgrowth, and OECs movement (Giacobini et al., 2004). Analyses of total number of GnRH cells and OECs, as well as the distance cells had migrated from the tip of the midline cartilage, were determined from stained images taken on a Nikon Eclipse E800 (Nikon USA, Melville, NY, United States) equipped with an ICCD camera (Retiga, Qimaging, Burnaby, Canada). The digital acquisition was made through IPLab software (IPLab Spectrum, Scanalytics Inc., Rockville, MD, United States) and analyzed with NIH ImageJ Software (Wayne Rasband)^1^. Distances of cell movement are expressed as cumulative frequency distributions for comparison. The control and treatment groups were then compared with a two-way ANOVA to test for differences in their cumulative frequency distributions; ANOVA data were considered significantly different if *p* < 0.005.

#### Live Imaging

To determine acute changes in GnRH neuronal migration rates, live imaging of was performed as described previously (Hutchins et al., 2016). This experiment consisted of 4–9 explants/treatment, from multiple culture dates, exposed to TX14A or MAC+TX14A. At 4 div, explants were placed into a chamber (Warner Instruments) maintained at 37 ± 2°C with 5% CO_2_ (Live cell; Pathology Devices, Inc.). Cells were identified based on morphology and location, and then later confirmed *post hoc* using immunostaining for GnRH. Cell positions were recorded every minute for 45 min for two periods. The first period was always just SFM. If using a single pharmacological agent, image acquisition for the second period started directly after the SFM period. However, for antagonist + agonist experiments, the antagonist was applied alone for a 15 min incubation period after the SFM period and then antagonist + agonist added, and second period recording started. Cell movement was measured, as previously described (Casoni et al., 2012; Hutchins et al., 2013). The position of the cell center was determined by the x and y coordinates plotted in ImageJ for the first and last frame for each individual cell. Rate was derived from the distance between the first and last positions divided by the total time (45 min) for each period. The first period defined the control for the treatment. Live imaging data were thus compared by paired *t*-test (Casoni et al., 2012; Hutchins et al., 2013) and considered significantly different if *p* < 0.001. All Statistical comparisons of data were calculated with Prism 7 (GraphPad).

### Analysis of the Olfactory Bulbs and GnRH Neurons in WT and GPR37 KO Littermates

The size of the olfactory bulbs and forebrains from GPR37 KO and WT E18.5 and adult mice was calculated by counting the number of 20 μm section taken through each structure. The area of the olfactory bulb and cellular layers in the olfactory bulbs of WT and KO littermates were also compared. Staining was performed for peripherin (E18.5), reelin (adult) or olfactory marker protein (OMP, adult) and Sox10 (both ages), and counterstained with Nissl, methyl green or toluidine blue as described above. Images were taken, digitized and analyzed using ImageJ. The thickness of olfactory nerve layer was determined using Reelin/Nissl to demarcate the structure and peripherin (or OMP in adult) staining to identify the nerve layer. The thickness of glomerular layer and mitral cell and external plexiform layer were measured using Nissl staining (on Reelin immunostained sections) for landmarks. Sox10 positive signals were quantified using ImageJ via threshold and the optical density of area measured within the nerve layer. Specifically, pictures were taken at 20x, and converted to 8 bit black and white format. A 300-pixel by 600-pixel region of interest (ROI) in E18.5 nerve layer or a 300-pixel by 550-pixel ROI in adults nerve layer were selected. Sox10 signals were then analyzed by adjusting the threshold, and signal intensity was measured and recorded in ImageJ for each selected area. Data of WT and KO animals were compared using a One-Way ANOVA. At least three animals in each genotype and age were used for the analysis. Significance was defined as *p* < 0.05.

GPR37 WT and KO animals were cut (serial non-fixed sections, parasagittal for embryos and coronal for adult brain, 18–20 μm thickness) and immunocytochemically stained for GnRH as described above. The total number of GnRH neurons at E11.5 was determined. The total number and distribution of GnRH neurons were quantified at E12.5, E15.5, E18.5, and adulthood (≥PN56). For distribution analysis GnRH cells were categorized by specific anatomical regions relevant to the age. At E12.5, regions included the vomeronasal organ (VNO), axon-tracks, and nasal brain junction (NBJ). At E15.5 and E18.5, regions included the nose, NBJ/olfactory bulbs (OB), and brain. In the adult brain, GnRH neuronal numbers were quantified as a function of distance from the organum vasculosum of lamina terminalis (OVLT). Total cell number was compared using a paired *t*-test at E11.5 since there was variation in the size of the embryos across litters at this embryonic stage. At all other ages, total GnRH cell number was compared using a One-way ANOVA (significance *p* < 0.05). Data for the GnRH cell distribution in the embryos were compared using a two-way ANOVA (significance is defined as *p* < 0.05) and *post hoc* analysis was performed using Sidak’s multiple comparison test, while the distribution of GnRH cells in adult brains were compared using the Kolmogorov-Smirnov (KS) test. All data are expressed as mean ± SEM.

## Results

GnRH neurons migrate along vomeronasal axons, in association with OECs, from the nasal placode to the forebrain during prenatal development. In mice these events occur between embryonic day (E) 10.5–E14.5, with the majority of GnRH cells within the forebrain by E14.5.–E16.5 (Wray, 2010). To evaluate the functional significance of GPR37 signaling during development of the GnRH/olfactory systems, the spatiotemporal expression pattern of GPR37 and its ligand, PSAP, was determined in cells in the nasal region.

### GPR37 and PSAP Are Expressed in GnRH Cells and OECs During Development

**FIGURE 1:**
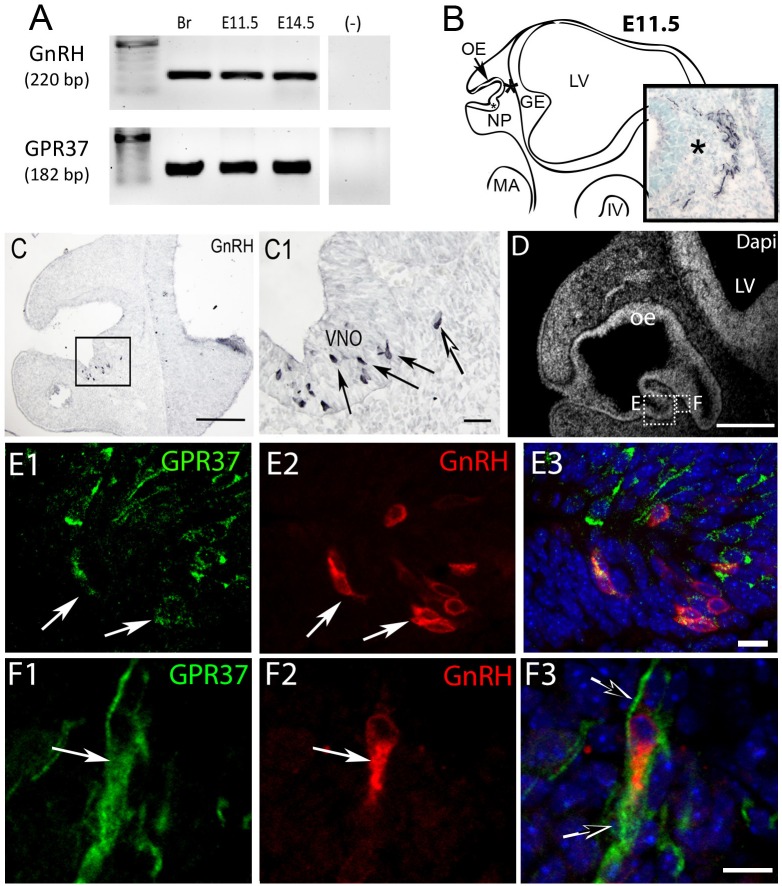
GPR37 is expressed in developing GnRH neurons. **(A)** GPR37 transcript (182 bp) was detected in Adult brain (Br), E11.5 nose, and E14.5 nose. Positive control GnRH transcript (220 bp); Negative control water (-). **(B)** Schematic of E11.5 embryo (NP, nasal pit; OE, olfactory epithelium; GE, ganglion eminence; LV, lateral ventricle; MA, mandibular process; IV, fourth ventricle) showing location of cells expressing GPR37 in the migratory mass (blue black staining photomicrograph inset; same location in schematic and inset indicated by asterisk). Cells in the migratory mass originate in the nasal placode. **(C)** Chromogen immunocytochemistry on E11.5 mouse sections stained for GnRH. GnRH expression is robustly detected in cells in the nasal pit (black arrows in **C1** which is boxed area of **C**) as well as cells that have started to migrate (white arrow in **C1**). **(D–F)** Immunofluorescence staining for GPR37 (green) GnRH (red) and Dapi (white in **D**, blue **E3**,**F3**). **(D)** Low magnification showing nasal area from which E and F are taken. **(E,F)** Low levels of GPR37 were detected in GnRH cells in the nasal pit (**E**, white arrows), while stronger GPR37 staining was detected in migrating GnRH cells (**F**, white arrow), as well as other elements associated with the migration route (**F3**, black arrows). Scale bars: **(C,D)** = 100 mm, **(C1,D1)** = 50 μm, **(E,F)** = 10 μm.

**FIGURE 2:**
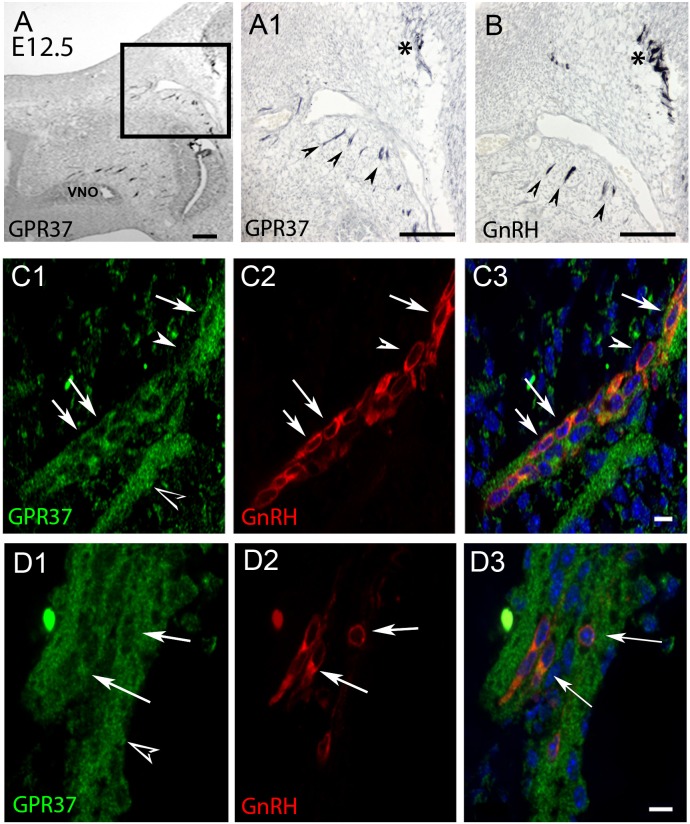
GPR37 expression increases in nasal region at E12.5. and overlaps with GnRH cell expression pattern. Consecutive sections **(A,B)** show GPR37 expression (**A1**: higher magnification of boxed region in **A**) in cells in tracts (arrowheads) that coincide with the tracts that GnRH cells (**B**, arrowheads) use to migrate to the nasal forebrain junction (**A1,B**, asterisk). Double label immunofluorescence confirms that GPR37 (green) is expressed in GnRH cells (red) in the migratory tracks **(C)** and nasal forebrain junction **(D)**. White arrows indicate double-labeled cells (**C,D**, merged image is shown in **C3,D3**). However, some GnRH positive cells are GPR37 negative (**C**, arrowhead) and GPR37 staining appears in elements surrounding the GnRH cells (**D1**, black arrowhead). Scale bar: **(A)** = 40 μm, **(A1,B)** = 100 μm, **(C,D)** = 10 μm.

**FIGURE 3:**
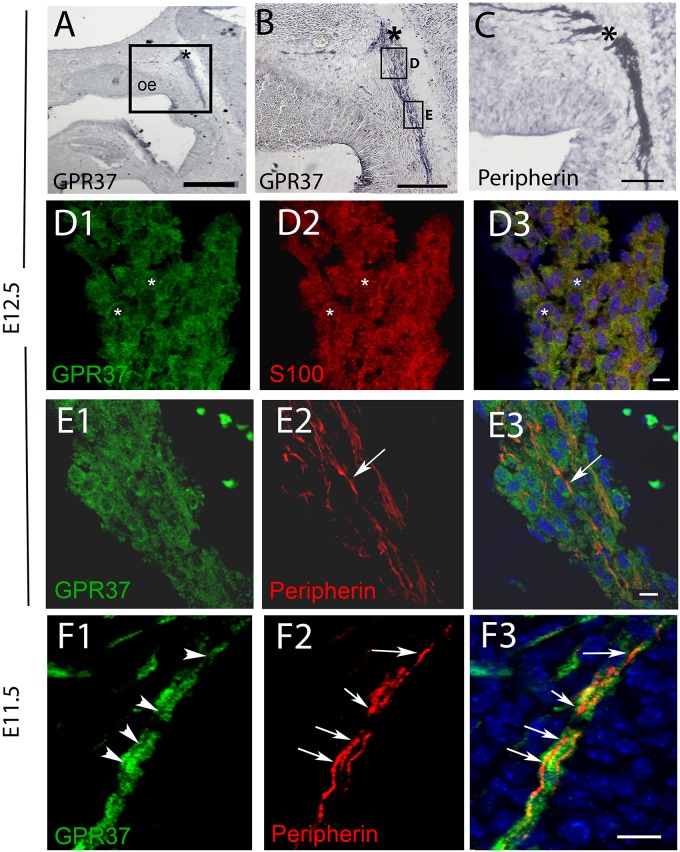
GPR37 is expressed in OECs but not in OSAs. **(A,B)** GPR37 expression detected in migratory tracks and nasal forebrain junction (^∗^) coincides with regions stained for OSA (peripherin staining, **C**). However, immunofluorescent staining showed GPR37 (green) colocalized with OECs (red, **D**, S100, same cells shown with small white asterisks) but not with OSAs **(E,F)**. **(E)** Arrow highlights single peripherin fiber that is GPR37 negative at E12.5 **(E)**. GPR37 positive elements are likely OECs which surround OSAs (see schematic Supplementary Figure 3). **(F)** Arrows **(F2,F3)** point to OSAs (red) that are GRP37 negative, shown in (**F1)** (arrowheads) at E11.5. **(D3–F3)** Merged images of corresponding row. **(A–G)**, E12.5; **(H)** E11.5. Scale bar: **(A)** = 200 μm, **(B,D)** = 100 μm, **(E)** = 20 μm, **(F)** = 10 μm. See Supplementary Figure 2 for surface rendering image.

**FIGURE 4:**
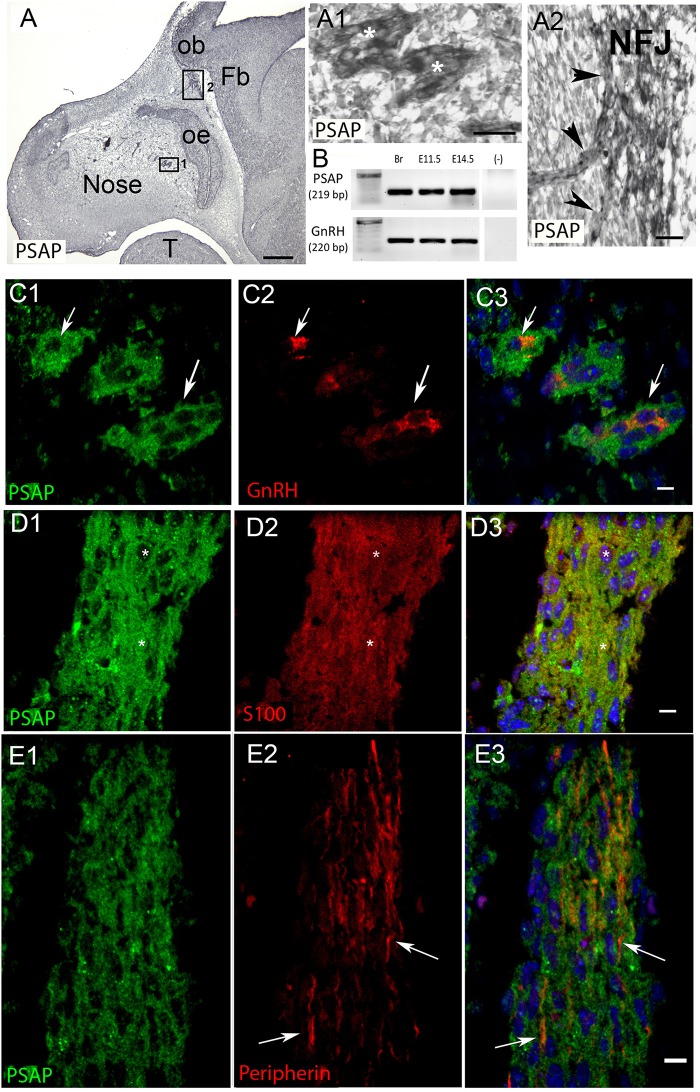
PSAP is expressed in GnRH neurons and OECs in the developing nasal region. **(A)** Immunocytochemistry for PSAP in a E12.5 embryo shows positive signal in tracts (**A1**, high magnification of boxed area 1 in **A**, asterisks) that converge at the nasal forebrain junction (**A2**, high magnification of boxed area 2 in **A**, NFJ). **(B)** PCR confirmed PSAP transcripts in nose region from E11.5 and E14.5 embryos [positive control brain (Br), negative control water (-), tissue control PCR for GnRH transcripts]. **(C–E)**. PSAP (green) colocalized with GnRH neurons (red, arrows **C**) and OECS (red, **D**), but was not present on OSAs (red, arrows **E**). All sections are from a E12.5 embryo. Panel 3 is merged image of corresponding row. Scale bar: **(A)** = 250 μm, **(A1,A2)** = 25 μm **(C–E)** = 10 μm.

PCR confirmed GPR37 expression in cDNA made from E11.5 and E14.5 noses (Figure 1A). At E11.5, a small group of intensely stained GPR37 immunopositive cells were detected within the nasal mesenchyme close to the nasal/forebrain junction (Figure 1B, inset). These immunopositive cells appeared to be in the migratory mass, composed of neurons and OECs (Forni et al., 2013), which is known to originate from the nasal placode. At this stage, most GnRH cells are located within the developing vomeronasal organ (VNO, Figure 1C,C1). Double label immunofluorescence indicated a few GnRH+/GPR37+ cells within the VNO (Figure 1E, white arrows, location of E shown in Figure 1D). At E11.5, a small number of GnRH cells are found outside the VNO, migrating toward the forebrain (Figure 1C, white arrow), and these were GPR37 positive [Figure 1F (white arrow), location of F shown in Figure 1D]. Additional elements along the tracts were clearly GRP37 positive (Figure 1F3, black arrows). A similar pattern for GPR37 was detected between E12.5 (Figure 2) and E14.5 (data not shown), with strong GPR37 staining (Figure 2A) detected along the GnRH cell migratory route (Figure 2B), both in the tracks (Figure 1A, 2B arrowheads) and at the nasal forebrain juncture (Figure 2A1,B, asterisk). The majority of GnRH cells on these tracks co-labeled with GPR37 (2C, arrows). Colocalization was maintained until the nasal forebrain junction (Figure 2D, arrows, asterisk Figure 2A for location). Not all GnRH cells co-labeled (Figure 2C, arrowhead). In addition, robust GPR37 staining was also present on non-GnRH elements in the migratory track (Figure 2C1, black arrowhead) and at the nasal forebrain junction (Figure 2D1, black arrowhead). Since GnRH cells migrate in association with OECs along olfactory axons, GPR37 expression in OECs (marked by Sox10 and S100, Au and Roskams, 2002; Forni and Wray, 2012) and sensory axons (marked by peripherin, Kramer et al., 2000) was examined at E12.5, when both axons and OECs are robust in the migratory tracts (Figure 3). GPR37 was found co-expressed on OECs (Figure 3D) but not olfactory axons (Figure 3E). GPR37 expression in olfactory axons was also examined at E11.5 (Figure 3F and Supplementary Figure 2) and E14.5 (data not shown), and co-expression was not detected. Note: GPR37 negative “channels” were detected in GPR37 positive tracks (Figure 3F1, arrowheads and Supplementary Figure 2). These “channels” contained peripherin positive fibers (Figure 3F2, arrows), that if coursing over a GnRH cell or under an OEC, both positive for GPR37, can give a false positive, i.e., the appearance of double-labeling (Supplementary Figure 3). The staining for the ligand, PSAP, mimicked the staining pattern found for the receptor (Figure 4). PSAP transcript was present in E11.5 and E14.5 noses (Figure 4B). PSAP expression was detected in the migratory mass, at E11.5 (data not shown), and in the migratory tracks, at E12.5 (Figure 4A,A1 asterisk) leading to the nasal forebrain junction (Figure 4A2, arrowheads, NFJ). Immunofluorescence double labeling colocalized PSAP in GnRH neurons (Figure 4C), and OECs (Figure 4D) but not sensory axons (Figure 4E).

**FIGURE 5:**
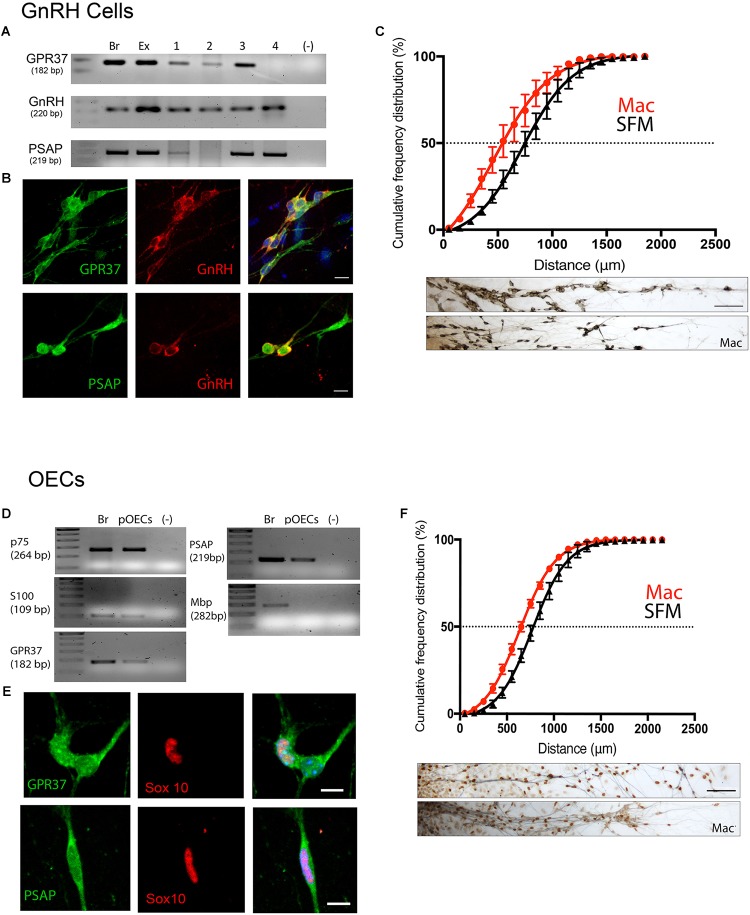
Inhibition of GPR37 in nasal explants attenuates migration of GnRH cells and OECs. **(A–C)** Analysis of GnRH cells. **(D,E)** Analysis of OECs. GPR37 and PSAP are expressed in migrating GnRH neurons and OECs as determined by PCR **(A,D)** and immunofluorescent staining on 4DIV explants **(B,E)**. Chronic application of Macitentan (Mac, a GPR37 antagonist) decreases migration of GnRH cells **(C)** and OECs **(F)**. Migration data presented as the cumulative frequency of Mac treated groups (red) vs. SFM groups (black), and indicate that Mac treatment reduced (shifted to the left) the distance both GnRH cells and OECs moved. Panels below each histogram show examples of stained GnRH/OECs (GnRH and Sox10, brown) and olfactory sensory fibers (pheripherin, blue). Statistics for measurements: GnRHs, *N* = 6 explants per group. For OECs, *N* = 11 and *N* = 14 explants for control and treatment groups. Scale bar: **(B,D)** = 10 μm, **(C,F)** = 50 μm.

To determine the function of GPR37 in the developing olfactory/GnRH systems an *in vitro* model, nasal explants, and GRP37 KO mice were used. The mouse nasal explant model maintains a large population of GnRH cells, olfactory sensory cells and OECs that migrate and extend in a manner similar to that reported for the nasal region *in vivo* (Fueshko and Wray, 1994; Raucci et al., 2013). This model allows for specific pharmacological experiments, with either acute or chronic exposure and measurements of cell migration and axon outgrowth (Dairaghi et al., 2018). GPR37 and PSAP expression was confirmed in GnRH neurons and OECs maintained *in vitro* via PCR (Figure 5A,D) and immunofluorescence (Figure 5B,E). As *in vivo*, GPR37 (Supplementary Figure 3) and PSAP (data not shown) were not present on olfactory sensory axons. Since endogenous levels of PSAP were present in the explants, Macitentan (Mac), a known antagonist of GPR37, or vehicle, was chronically applied.

**FIGURE 6:**
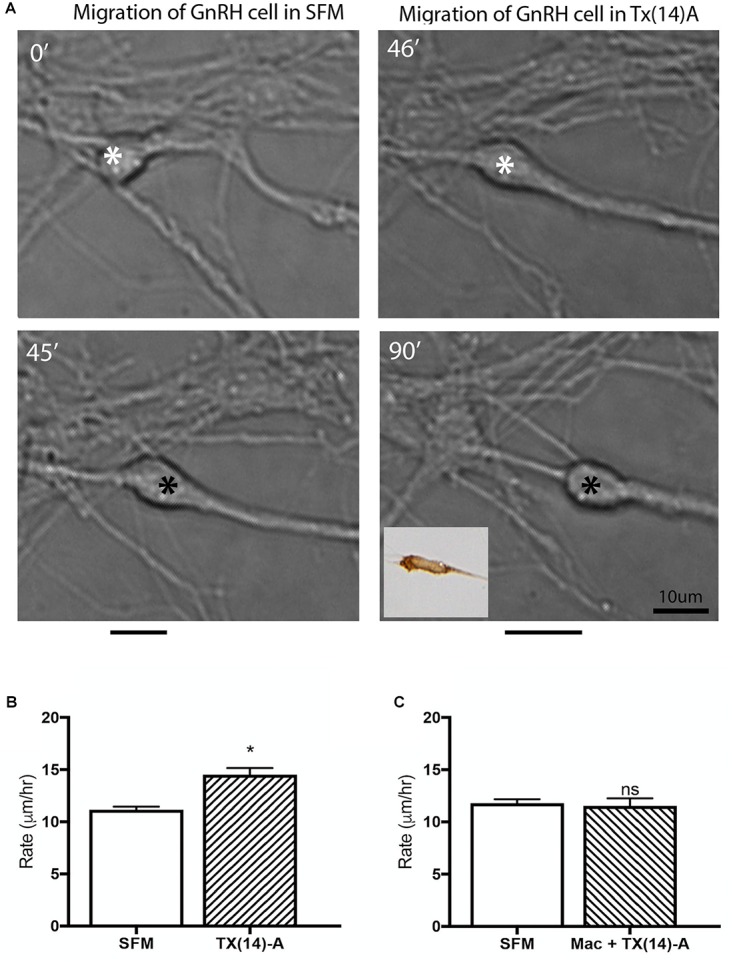
Acute application of TX(14)A accelerates GnRH migration rate in nasal explants. **(A)** Example of acute assay showing GnRH cell in pre-treatment condition (SFM 45 min) and posttreatment condition (TX14A 45 min). Asterisk indicates location in cell soma where measurements were taken. Black lines beneath pictures indicate the distance moved during that 45 min treatment period. Phenotype of cell images was confirmed as GnRH *post hoc* via immunocytochemistry (inset). Scale bar: 1 μm. **(B)** Histogram shows significant increase in migration rate of SFM/Tx14A (*N* = 9 explants, *n* = 225 cells, *p* < 0.0001). **(C)** No change in migration rate was observed after application of Mac+TX14A (*N* = 4 explants, *n* = 158 cells, *p* = 0.044).

GnRH cells in explants treated with Mac showed a dramatic reduction in the distance that the cells migrated into the periphery as compared to controls (*p* < 0.0001, Two-way ANOVA, *N* = 6 explants per group, Figure 5C). In addition, decreased migration of OECs (Figure 5F) and outgrowth of olfactory axons (Data not shown) were also found (OECs; *p* < 0.0001, Two-way ANOVA, *N* = 11 and *N* = 14 explants for control and treatment groups, respectively; olfactory axons: *p* < 0.0001, unpaired *T*-test, *N* = 17 and *N* = 20 explants for control and treatment group, respectively). Since both OEC migration and olfactory axon outgrowth were reduced with Mac treatment, the decrease in GnRH cell migration could have been an indirect effect, resulting from direct perturbation in OECs or an indirect perturbation of sensory axons. To examine this, acute migration assays were performed (Figure 6). GnRH cell movement was analyzed for a 1 h treatment period and compared to a 1 h control period. Treatment groups consisted of TX14A alone, a functional Prosaptide and ligand for GPR37 or Mac +TX14A. Addition of TX14A (1 μM, Tocris) significantly increased GnRH cell migration rate (*p* < 0.0001, paired *t*-test, *N* = 9 explants, 255 cells). The increase in migration rate with TX14A was abolished by the pretreatment and coapplication with the antagonist Mac (2 μM), *p* = 0.727, paired *t*-test, *N* = 4 explant, 158 cells). These acute assays are consistent with activation of the GPR37 receptor occurring directly on GnRH neurons. Note that the GnRH cell migration in the Mac + TX14 group was not significantly different from the control groups, consistent with Mac acting on GPR37 and not another pathway to reduce GnRH cell migration in the chronic study.

### Lack of GPR37 Delays GnRH Neuronal Migration and Reduces Olfactory Nerve Layer

**FIGURE 7:**
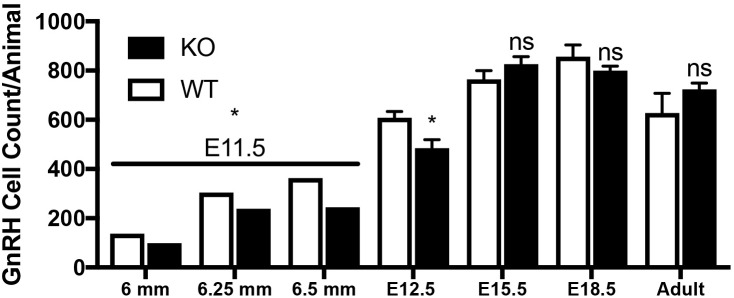
Lack of GPR37 delays GnRH development. Significantly fewer GnRH positive neurons were observed at E11.5 (N: 6 WT and 6 KO; *p* = 0.0034 paired *t*-test) and E12.5 (N: 3 WT and 4 K, *p* = 0.041 paired *t*-test). By E15.5 (N: 4 WT and 4 KO), and thereafter (E18.5, N: 3 WT, 4 KO, and Adult, N: 3 WT and 3 KO), no differences in the number GnRH neurons was detected (*p* = 0.231, *p* = 0.266, *p* = 0.315, respectively, One way-ANOVA). ^∗^*p* < 0.05, ns, not significant, *p* > 0.05.

GPR37^+/-^ mice were time-mated and embryos and adult brains were collected, and the number and location of GnRH neurons quantified (Figure 7, E11.5: *N* = 4 WT and KO, E12.5: *N* = 3 WT, *N* = 4 KO, E15.5: *N* = 4 WT and KO, E18.5: *N* = 3 WT, *N* = 4 KO, adult brains: N = 3 WT and KO).

**FIGURE 8:**
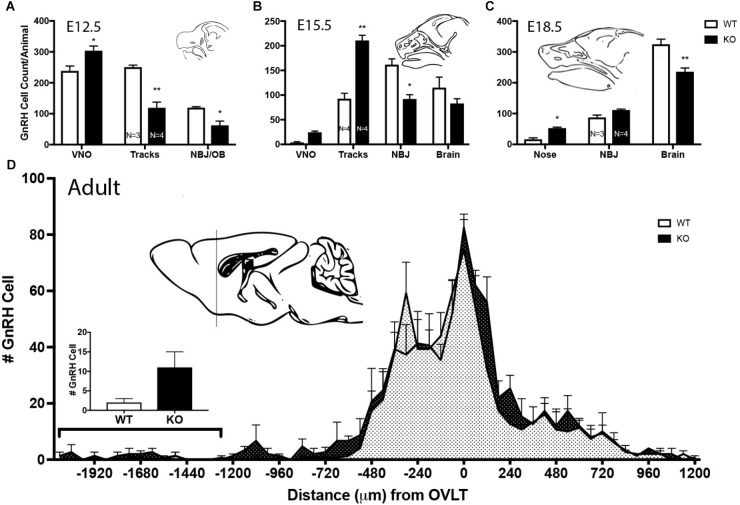
Lack of GPR37 delays GnRH cell migration. **(A–C)** Distribution of GnRH cells at indicated embryonic ages. Cell location in embryos was classified depending of age; 3 regions at E12.5 [vomeronasal organ (VNO), migratory tracts, and nasal forebrain junction (NFJ)/olfactory bulb (OB)], 4 regions at E15.5 (VNO, tracks, NFJ, and Brain), and 3 regions at E18.5 (Nose, NFJ and Brain). At all three embryonic ages, there was a significant area × genotype interaction (*p* < 0.0001, Two-way ANOVA), indicating the two genotypes behaved differently. *Post hoc* analysis (Sidak’ multiple comparison test, ^∗^*p* < 0.05, ^∗∗^*p* < 0.001) revealed a general trend across ages, with the KO mice having more GnRH cells in the nasal regions (VNO, Tracks, and Nose) and less cells in the brain compared to WT littermates. **(D)** In the adult brain, GnRH cells were mapped relative to the OVLT (negative numbers are toward the olfactory bulbs). KO animals have more GnRH cells in anterior regions. Histogram indicates number of GnRH cells from -1200 (line of schematic on sagittal brain section) to -1920. GnRH cells remained more rostral in the KO as compared to the WT (KS Test, *p* = 0.027; *N* = 3 for both groups).

Mutants had significantly less GnRH cells at E11.5 and E12.5 compared to WT littermates (E11.5 *N* = 3 each: WT = 250 ± 55, KO = 194 ± 77, *p* < 0.05, paired *t*-test; E12.5 *N* = 3 WT, 4 KO: WT = 608 ± 45, KO = 485 ± 67, *p* < 0.05, paired *t*-test). No difference between genotypes was found in the total number of GnRH cells later in development, i.e., at E15.5, E18.5 or in adult brains (Figure 7, *p* > 0.05 One-way ANOVA). The distribution of GnRH cells was quantified in E12.5, E15.5, and E18.5 embryos (Figure 8). At all three ages, there was a significant area x genotype interaction (*p* < 0.05, Two-way ANOVA), indicating the two genotypes behaved differently. *Post hoc* analysis revealed a general trend across ages, with the KO mice having more cells in the nasal regions (VNO and tracks) and less cells in the brain compared to WT littermates. These data indicate a delay in GnRH neuronal migration occurred in GPR37 KOs. Even in adult brains, more GnRH cells were in rostral regions in KO compared to WT mice, consistent with a delay in GnRH migration (*p* = 0.021, KS test, *N* = 3 for both genotypes, Figure 8).

**FIGURE 9:**
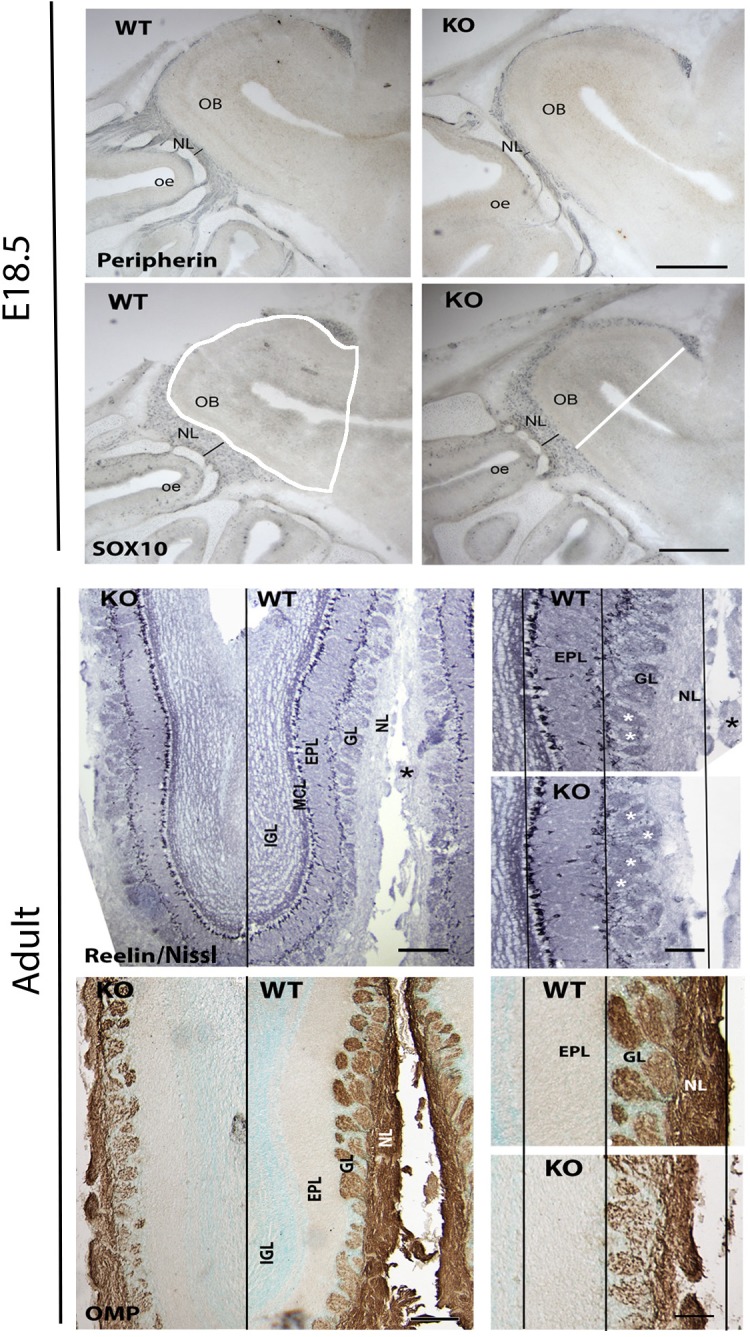
Absence of GPR37 impacts olfactory nerve layer development. Upper panels show examples of immunocytochemical staining from E18.5 WT and KO embryos stained (blue) for peripherin and Sox10, markers for axons and OECs in the olfactory nerve layer. A significant decrease in the olfactory nerve layer (NL) thickness (black line) was found (Sox10 staining decreased 29% (WT = 133.5 ± 7.8 μm, KO = 95.3 ± 6.4 μm, *N* = 7/genotype, *p* = 0.003)] and (peripherin staining decreased 47% (WT = 100.2 ± 6.9 μm, KO = 53.4 ± 3.1 μm, *N* = 4/genotype, *p* = 0.0008)]. Lower panels show examples of adult olfactory bulbs from GPR37 WT and KO mice stained with Nissl to delineate the layers and OMP staining to mark the olfactory nerve layer. Left: low magnification of coronal sections. Black line separates KO from WT. Higher magnification of each shown on right (white asterisk = glomeruli). Black lines are drawn through the mitral cell layer (MCL), outer aspect of the external plexiform layer (EPL) and the outer aspect of the NL using the dimensions of the WT tissue. A reduction in the ONL was found in KO mice. GL, glomerular layer. Scale: top panel = 400 μm, left bottom panel = 200 μm, right bottom panel 100 μm.

Since GPR37 was detected in OECs during development, olfactory bulbs were examined at E18.5 and adult ages in KO mice (Figure 9) using Sox10 staining for OECs, peripherin (E18.5) and OMP (adult) for the olfactory nerve and general morphology using Nissl staining. Olfactory bulb area (Figure 9 WT Sox10 panel, white shape), cross-sectional diameter (Figure 9 KO Sox10 panel, white line), and the thickness of the olfactory nerve layer, glomeruli layer, and external granular layer were analyzed, comparing KO mice to WT littermates. At E18.5, there was 17.5% decrease in the overall area of the olfactory bulb in KO mice (*N* = 8/genotype, 1008 × 10^3^ μm^2^ vs. 1221 ×10^3^ μm^2^; *p* = 0.001) while the diameter of the olfactory bulbs was similar (WT = 1.174 ± 0.02 mm; KO = 1.128 ± 0.03 mm). Specific decreases were seen in the thickness of the olfactory nerve layer delineated by (1) Sox10 staining (decreased 29%, WT = 133.5 ± 7.8 μm, KO = 95.3 ± 6.4 μm, *N* = 7/genotype, *p* = 0.003), and (2) peripherin staining (decreased 47%, WT = 100.2 ± 6.9 μm, KO = 53.4 ± 3.1 μm, *N* = 4/genotype, *p* = 0.0008). No significant difference was found in the relative density of Sox10 staining (decreased by 22%, *p* = 0.22) nor in the width of glomerular layer at this age (WT = 93.7.5 ± 6.2 μm, KO = 76.6 ± 6.3 μm, *N* = 7/genotype, *p* = 0.08). In adult mice, measurements on Nissl sections included the width of the: (1) mitral cell and external plexiform layer, (2) glomerular layer, and (3) nerve layer. Both the glomerular layer and nerve layer were significantly decreased in KOs compared to WT littermates (GL: WT = 237 ± 41 μm; KO = 179 ± 19 μm, *p* = 0.043; NL: WT = 154 ± 10.5 μm; KO = 99.6 ± 23.6 μm, *p* = 0.0056), with the glomerular layer decreasing by 24% and the nerve layer by 35%. No change was detected in the mitral/external plexiform layer (WT = 266.8 ± 57.5 μm, KO = 260.8 ± 52.5 μm, *p* = 0.8831). To verify changes in the nerve layer and examine the presence of OECs, measurements were obtained from sections stained for OMP and Sox10, respectively. Significant differences between genotypes were detected using both these markers. OMP staining showed that the nerve layer decrease by 34% (*p* = 0.035, WT = 131.8 ± 11 μm, KO = 87.35 ± 8.5 μm), in agreement with the value obtained from the Nissl sections. The change in the relative amount of Sox10 staining in the nerve layer decrease by 44% (*p* = 0.007). Note: the overall length of WT and KO adult brains was calculated from cut sections. No differences were detected between genotypes in either the forebrain (from behind the olfactory bulbs to the caudal hypothalamus; *p* = 0.826, WT = 5.5 ± 0.139 mm; KO = 5.52 ± 0.139 mm) or olfactory bulbs (WT = 2.96 ± 0.35 mm; KO = 2.8 ± 0.174 mm). In addition, the width of the brains was similar at the crossing of the anterior commissures (*p* = 0.24: WT = 8.27 ± 0.37 mm; KO = 8.83 ± 0.167 mm). Thus, the detected changes are not the results of overall changes in brain size.

## Discussion

GPR37 has been implicated in the pathophysiology of Parkinson’s Disease (Smith, 2015), modulation of dopamine signaling (Marazziti et al., 2004), and Sertoli cell maturation/proliferation (La Sala et al., 2015), but little is known about its function in the development of the nervous system. Recently, GPR37 was shown to be an ER chaperone for LRP6 and required to maintain normal LRP6 protein levels and Wnt/β-catenin signaling, as LRP6 is an essential Wnt co-receptor in neural progenitor cells to promote Wnt-dependent neurogenesis (Berger et al., 2017) and migration. In this study, we characterized the expression of GPR37 in the developing GnRH/olfactory system and identified a novel role for PSAP-GPR37 signaling on GnRH and OEC migration and neurogenesis. Using *in vivo* and *in vitro* assays, we show that PSAP signals via GPR37 drive OEC migration to the olfactory bulb and GnRH neuronal differentiation and migration into the CNS.

PCR and immunofluorescence confirmed GPR37 and Prosaposin expression in OECs during embryonic development. Functional studies showed that blocking GPR37 attenuated OEC migration and olfactory axon outgrowth in nasal explants. Analysis of GPR37 KO mice, revealed that the olfactory nerve layer, consisting of olfactory axons and OECS is reduced both prenatally and in the adult. It is known that olfactory axons prefer to grow on OECS (Raucci et al., 2013) and that defective olfactory axon targeting and thinner olfactory nerve layers occur when OEC differentiation is disrupted (Forni et al., 2011; Barraud et al., 2013). Our data suggest that signaling through GPR37 is involved in this interaction and olfactory axons are indirectly altered by disruption of GRP37 in OECs.

*GPR37* is localized on chromosome 7q31–33, called the *AUTS1* region, the first locus linked to autism (Alarcon et al., 2002; Auranen et al., 2002), and has been identified in 2 patients with autism spectrum disorder (ASD, Fujita-Jimbo et al., 2012). Hyper- or hypo- reactivity to sensory stimuli, including over-smelling, is now considered in the criteria for ASD diagnosis. A number of studies have documented olfactory dysfunction in ASD (Tonacci et al., 2017). Specifically, hyposensitivity was found, consistent with a deficit in the peripheral olfactory system, i.e., a higher concentration of the stimulus was needed to be detected (Dudova et al., 2011; Muratori et al., 2017). No data is available on the olfactory function of the ASD patients with *GPR37* mutations. However, work on GPR37 has been related to Parkinson’s disease. Parkinson’s disease, long known as a “movement” disorder, has many “non-motor” deficits including olfaction function (Chaudhuri et al., 2005; Langston, 2006; Chaudhuri and Odin, 2010). Mandillo et al. (2013) extended the behavioral characterization of GPR37 KO mice to olfaction. Using the buried food test, they found that GPR37 KO mice were significantly slower to find food than their WT littermates (Mandillo et al., 2013), suggesting a decrease in general olfactory function. Notably, aged KO mice spent more sniffing time in the habituation/dishabituation test, at both the last habituation test and novel scent test, than WT littermates. The authors suggest this is indicative of better odor detection in the KO compared to WT. However, the KO mice responded with the same sniffing time at all trials in this test, longer than WT, which could indicate decreased olfactory function. Independent of interpretation, the study by Mandillo et al. (2013) indicates that olfactory function changed in adult GPR37 KO mice and are consistent with the anatomical changes observed in the present study. Together, these data support GPR37 signaling in OECs modulates sensory neuron axonal outgrowth and as such, olfactory bulb development.

GPR37 was expressed in GnRH cells as they left the developing VNO and migrated to the brain. Similar results were found for GPR37’s ligand Prosaposin. To address the function of GPR37 in GnRH neurons, nasal explants and KO mice were used. Nasal explants maintain a large population of GnRH neurons that migrate in a similar fashion to GnRH neurons *in vivo* (Fueshko and Wray, 1994). Chronic application of the antagonist attenuated GnRH neuronal migration. However, since OEC migration was also perturbed, the change in GnRH neuronal migration could have been indirect. To determine if a direct effect of PSAP-GPR37 signaling on GnRH neurons was present, acute migration assays were used. Application of a GPR37 agonist [Prosaptide (TX14A), Meyer et al., 2013] resulted in a rapid increase in GnRH neuronal migration rate, which was not observed with prior blocking of GPR37 with Macitentan. These results support a direct effect of Prosaposin/GPR37 signaling on GnRH cell movement. The role for GPR37 signaling in GnRH neuronal migration was further evaluated *in vivo* using GPR37 KO mice. GnRH neurons were “slowed” along their migrational route at E12.5 and E18.5, remaining closer to their site of origin (the VNO) in GPR37 KO mice and was observed in the final GnRH distribution in adults; although many GnRH neurons had reached their appropriate destination, a significant increase was found in those that remained rostral, close to their entry point at the nasal forebrain junction. Together, these data make signaling via PSAP/GPR37 a modulator of GnRH neuronal migration.

Unexpectedly, at E11.5 and E12.5, there were fewer GnRH neurons in GPR37 deficient embryos compared to their WT littermates. A subpopulation (30%) of GnRH cells in mice has been proposed to be derived from neural crest (Forni and Wray, 2012). At E11.5, GPR37 KOs had 22% less GnRH cells in the VNO. The loss of cells was still present at E12.5 (20%) but was not detected by E15.5. Notably a role for GPR37 in Wnt dependent neurogenesis, where a proposed delay in neurogenesis is due to the absence of GPR37, has been suggested (Berger et al., 2017). Whether this accounts for the delay in the subpopulation of GnRH cells detected remains to be determined. Canonical Wnt signaling, which requires GPR37, is also involved in establishing olfactory axon connections into the forebrain, affecting formation of the nerve layer in the OB (Zaghetto et al., 2007). Perturbation of GPR37 in mutant animals resulted in decreased thickness in nerve layer of olfactory bulbs, further suggesting that interplay of GPR37 and Wnt signaling together are important factors in olfactory development.

OECs are also neural crest derived (Barraud et al., 2010; Forni et al., 2011). Although too numerous to count, we cannot rule out that a lower OEC number contributes to the decrease in SOX10 measurement detected in the olfactory bulbs. Previously, it has been suggested that GPR37L1 and Mesoderm development (Mesd) may compensate for GPR37 loss of function (Berger et al., 2017). Evaluating our microarray data *post hoc* for these 2 genes in GnRH cells, showed that GPR37L1 is not highly expressed. However, Mesoderm development candidate 1 and 2 are robustly expressed. Mesd is an important regulator of Low-density lipoprotein receptor related protein six folding and thus the canonical Wnt/β-catenin signaling pathway (Gray et al., 2013). This pathway is involved in lineage specification, with high Wnt biasing neural crest cells toward neuronal differentiation and low Wnt toward mesenchymal derivatives (Hari et al., 2012). Further experiments are required to determine if Mesd is involved in compensating in neural crest development in GRP37 KOs which results in (1) an increase in the number of GnRH cells detected between E12.5 and E15.5 and/or (2) maintaining OEC cell number. In summary, the experiments in this report clearly show that PSAP-GPR37 signaling plays a role in the development of the GnRH/olfactory system. Recent studies show the existence and prevalence of oligogenism in Kallman Syndrome patients (Sykiotis et al., 2010), indicating that a combination of mutations or rare variants on two or more genes can underlie the disease (di/oligogenic disorder). As such, mutations in Gpr37 are candidates when searching for oligogenism associated with Kallman’s Syndrome.

## Ethics Statement

All procedures were approved by National Institute of Neurological Disorders and Stroke (NINDS) ACUC and performed in accordance with National Institutes of Health (NIH) guidelines.

## Author Contributions

HS and SW designed the research. HS and YS performed the research. DM for mouse line. HS, YS, and SW analyzed the data and wrote the manuscript.

## Conflict of Interest Statement

The authors declare that the research was conducted in the absence of any commercial or financial relationships that could be construed as a potential conflict of interest.

## Footnotes

1 http://rsbweb.nih.gov/ij/

## Supplementary Material

FIGURE S1 Specificity of GPR37 antibody. Double-label immunofluorescence on explants generated from WT and KO E11.5 embryos, maintained for 4 days in culture, fixed and stained for GPR37 (red, rabbit antibody) and GnRH (green, mouse antibody). A no primary control was run at the same time. Neither the KO nor the no primary control showed a red GPR37 signal. Single label immunofluorescence for GRP37 (green) was run on adult olfactory bulb sections from WT and KO littermates. A no primary control was run at the same time. GPR37 is only seen in the WT OB section, particularly in the olfactory nerve layer (ONL). G, glomeruli. Scale: Explant panels = 10 μm, adult panel: 50 μm.

FIGURE S2 GPR37 is not expressed by olfactory axons *in vivo*. Imaris generated surface rendering processed from confocal stack, one of which is shown in Figure 2 E11.5. Reconstruction shows GPR37 negative “channels” (arrowheads) in GPR37 positive tracks (green). These “channels” contained peripherin positive fibers (red, arrows). Scale: 30 μm.

FIGURE S3 GPR37 is not expressed by olfactory axons *in vitro*. **(A)** 4DIV explant stained for peripherin (red, mouse antibody), which marks olfactory axons and GPR37 (green, rabbit antibody). *In vitro*, as *in vivo*, migratory tracks are composed of dense fibers and cells (see **B** right panel). However, peripherin fibers (white arrow) and GPR37 fibers (black arrows) are not double-labeled. False positive can occur as shown in **(B)**. Left panel shows image of peripherin fiber (green) coursing over a GnRH cell (red). GnRH cells are not peripherin positive, yet yellow puncta are seen (arrowheads). Right panel shows schematic of the relationship between OECs which are GRP37 positive (green) and GnRH cells which are GPR37 positive (green) and peripherin fibers (red) which are GPR37 negative. As elements sit on each other false positives (orange) can occur. Scale: 10 μm.

## References

[B1] Alarcon M. Cantor R. M. Liu J. Gilliam T. C. Geschwind D. H. Autism Genetic Research Exchange Consortium (2002). Evidence for a language quantitative trait locus on chromosome 7q in multiplex autism families. *Am. J. Hum. Genet.* 70 60–71. 10.1086/338241 11741194 PMC384904

[B2] Au E. Roskams A. J. (2002). Culturing olfactory ensheathing glia from the mouse olfactory epithelium. *Methods Mol. Biol.* 198 49–54. 11951640 10.1385/1-59259-186-8:049

[B3] Auranen M. Vanhala R. Varilo T. Ayers K. Kempas E. Ylisaukko-Oja T. (2002). A genomewide screen for autism-spectrum disorders: evidence for a major susceptibility locus on chromosome 3q25-27. *Am. J. Hum. Genet.* 71 777–790. 10.1086/342720 12192642 PMC378535

[B4] Barraud P. Seferiadis A. A. Tyson L. D. Zwart M. F. Szabo-Rogers H. L. Ruhrberg C. (2010). Neural crest origin of olfactory ensheathing glia. *Proc. Natl. Acad. Sci. U.S.A.* 107 21040–21045. 10.1073/pnas.1012248107 21078992 PMC3000254

[B5] Barraud P. St John J. A. Stolt C. C. Wegner M. Baker C. V. (2013). Olfactory ensheathing glia are required for embryonic olfactory axon targeting, and the migration of gonadotropin-releasing hormone neurons. *Biol. Open* 2 750–759. 10.1242/bio.20135249 23862023 PMC3711043

[B6] Berger B. S. Acebron S. P. Herbst J. Koch S. Niehrs C. (2017). Parkinson’s disease-associated receptor GPR37 is an ER chaperone for LRP6. *EMBO Rep.* 18 712–725. 10.15252/embr.201643585 28341812 PMC5412897

[B7] Bouilly J. Messina A. Papadakis G. Cassatella D. Xu C. Acierno J. S. (2018). DCC/NTN1 complex mutations in patients with congenital hypogonadotropic hypogonadism impair GnRH neuron development. *Hum. Mol. Genet.* 27 359–372. 10.1093/hmg/ddx408 29202173

[B8] Casoni F. Hutchins B. I. Donohue D. Fornaro M. Condie B. G. Wray S. (2012). SDF, and GABA interact to regulate axophilic migration of GnRH neurons. *J. Cell Sci.* 125(Pt 21), 5015–5025. 10.1242/jcs.101675 22976302 PMC3533389

[B9] Chaudhuri K. R. Odin P. (2010). The challenge of non-motor symptoms in Parkinson’s disease. *Prog. Brain Res.* 184 325–341. 10.1016/S0079-6123(10)84017-8 20887883

[B10] Chaudhuri K. R. Yates L. Martinez-Martin P. (2005). The non-motor symptom complex of Parkinson’s disease: a comprehensive assessment is essential. *Curr. Neurol. Neurosci. Rep.* 5 275–283. 10.1007/s11910-005-0072-6 15987611

[B11] Dairaghi L. Flannery E. Giacobini P. Saglam A. Saadi H. Constantin S. (2018). Reelin can modulate migration of olfactory ensheathing cells, and gonadotropin releasing hormone neurons via the canonical pathway. *Front. Cell. Neurosci.* 12:228. 10.3389/fncel.2018.00228 30127721 PMC6088185

[B12] Donohue P. J. Shapira H. Mantey S. A. Hampton L. L. Jensen R. T. Battey J. F. (1998). A human gene encodes a putative G protein-coupled receptor highly expressed in the central nervous system. *Brain Res. Mol. Brain Res.* 54 152–160. 10.1016/s0169-328x(97)00336-7 9526070

[B13] Dudova I. Vodicka J. Havlovicova M. Sedlacek Z. Urbanek T. Hrdlicka M. (2011). Odor detection threshold, but not odor identification, is impaired in children with autism. *Eur. Child Adolesc. Psychiatry* 20 333–340. 10.1007/s00787-011-0177-1 21528391

[B14] Forni P. E. Bharti K. Flannery E. M. Shimogori T. Wray S. (2013). The indirect role of fibroblast growth factor-8 in defining neurogenic niches of the olfactory/GnRH systems. *J. Neurosci.* 33 19620–19634. 10.1523/JNEUROSCI.3238-13.2013 24336726 PMC3858631

[B15] Forni P. E. Taylor-Burds C. Melvin V. S. Williams T. Wray S. (2011). Neural crest, and ectodermal cells intermix in the nasal placode to give rise to GnRH-1 neurons, sensory neurons, and olfactory ensheathing cells. *J. Neurosci.* 31 6915–6927. 10.1523/JNEUROSCI.6087-10.2011 21543621 PMC3101109

[B16] Forni P. E. Wray S. (2012). Neural crest, and olfactory system: new prospective. *Mol. Neurobiol.* 46 349–360. 10.1007/s12035-012-8286-5 22773137 PMC3586243

[B17] Fueshko S. Wray S. (1994). LHRH cells migrate on peripherin fibers in embryonic olfactory explant cultures: an in vitro model for neurophilic neuronal migration. *Dev. Biol.* 166 331–348. 10.1006/dbio.1994.1319 7958456

[B18] Fujita-Jimbo E. Yu Z. L. Li H. Yamagata T. Mori M. Momoi T. (2012). Mutation in Parkinson disease-associated, G-protein-coupled receptor 37, (GPR37/PaelR) is related to autism spectrum disorder. *PLoS One* 7:e51155. 10.1371/journal.pone.0051155 23251443 PMC3520984

[B19] Giacobini P. Kopin A. S. Beart P. M. Mercer L. D. Fasolo A. Wray S. (2004). Cholecystokinin modulates migration of gonadotropin-releasing hormone-1 neurons. *J. Neurosci.* 24 4737–4748. 10.1523/jneurosci.0649-04.2004 15152034 PMC6729465

[B20] Gong S. Zheng C. Doughty M. L. Losos K. Didkovsky N. Schambra U. B. (2003). A gene expression atlas of the central nervous system based on bacterial artificial chromosomes. *Nature* 425 917–925. 10.1038/nature02033 14586460

[B21] Gray J. D. Kholmanskikh S. Castaldo B. S. Hansler A. Chung H. Klotz B. (2013). LRP6 exerts non-canonical effects on Wnt signaling during neural tube closure. *Hum. Mol. Genet.* 22 4267–4281. 10.1093/hmg/ddt277 23773994 PMC3792688

[B22] Hari L. Miescher I. Shakhova O. Suter U. Chin L. Taketo M. (2012). Temporal control of neural crest lineage generation by Wnt/beta-catenin signaling. *Development* 139 2107–2117. 10.1242/dev.073064 22573620

[B23] Howell G. R. MacNicoll K. H. Braine C. E. Soto I. Macalinao D. G. Sousa G. L. (2014). Combinatorial targeting of early pathways profoundly inhibits neurodegeneration in a mouse model of glaucoma. *Neurobiol. Dis.* 71 44–52. 10.1016/j.nbd.2014.07.016 25132557 PMC4319373

[B24] Hutchins B. I. Klenke U. Wray S. (2013). Calcium release-dependent actin flow in the leading process mediates axophilic migration. *J. Neurosci.* 33 11361–11371. 10.1523/JNEUROSCI.3758-12.2013 23843509 PMC3724331

[B25] Hutchins B. I. Kotan L. D. Taylor-Burds C. Ozkan Y. Cheng P. J. Gurbuz F. (2016). CCDC141 mutation identified in anosmic hypogonadotropic hypogonadism, (Kallmann Syndrome) alters GnRH neuronal migration. *Endocrinology* 157 1956–1966. 10.1210/en.2015-1846 27014940 PMC4870868

[B26] Imai Y. Inoue H. Kataoka A. Hua-Qin W. Masuda M. Ikeda T. (2007). Pael receptor is involved in dopamine metabolism in the nigrostriatal system. *Neurosci. Res.* 59 413–425. 10.1016/j.neures.2007.08.005 17889953

[B27] Klenke U. Taylor-Burds C. (2012). Culturing embryonic nasal explants for developmental, and physiological study. *Curr. Protoc. Neurosci.* 59 3.25.1–3.25.16. 10.1002/0471142301.ns0325s59 22470149 PMC3384499

[B28] Klenke U. Taylor-Burds C. Wray S. (2014). Metabolic influences on reproduction: adiponectin attenuates GnRH neuronal activity in female mice. *Endocrinology* 155 1851–1863. 10.1210/en.2013-1677 24564393 PMC3990841

[B29] Kramer P. R. Guerrero G. Krishnamurthy R. Mitchell P. J. Wray S. (2000). Ectopic expression of luteinizing hormone-releasing hormone, and peripherin in the respiratory epithelium of mice lacking transcription factor AP-2alpha. *Mech. Dev.* 94 79–94. 10.1016/s0925-4773(00)00316-6 10842061

[B30] Kramer P. R. Wray S. (2000). Midline nasal tissue influences nestin expression in nasal-placode-derived luteinizing hormone-releasing hormone neurons during development. *Dev. Biol.* 227 343–357. 10.1006/dbio.2000.9896 11071759

[B31] La Sala G. Marazziti D. Di Pietro C. Golini E. Matteoni R. Tocchini-Valentini G. P. (2015). Modulation of Dhh signaling, and altered Sertoli cell function in mice lacking the GPR37-prosaposin receptor. *FASEB J.* 29 2059–2069. 10.1096/fj.14-269209 25609427

[B32] Langston J. W. (2006). The Parkinson’s complex: parkinsonism is just the tip of the iceberg. *Ann. Neurol.* 59 591–596. 10.1002/ana.20834 16566021

[B33] Mandillo S. Golini E. Marazziti D. Di Pietro C. Matteoni R. Tocchini-Valentini G. P. (2013). Mice lacking the Parkinson’s related GPR37/PAEL receptor show non-motor behavioral phenotypes: age, and gender effect. *Genes Brain Behav.* 12 465–477. 10.1111/gbb.12041 23574697

[B34] Marazziti D. Golini E. Gallo A. Lombardi M. S. Matteoni R. Tocchini-Valentini G. P. (1997). Cloning of GPR37, a gene located on chromosome 7 encoding a putative G-protein-coupled peptide receptor, from a human frontal brain EST library. *Genomics* 45 68–77. 10.1006/geno.1997.4900 9339362

[B35] Marazziti D. Golini E. Mandillo S. Magrelli A. Witke W. Matteoni R. (2004). Altered dopamine signaling, and MPTP resistance in mice lacking the Parkinson’s disease-associated GPR37/parkin-associated endothelin-like receptor. *Proc. Natl. Acad. Sci. U.S.A.* 101 10189–10194. 10.1073/pnas.0403661101 15218106 PMC454186

[B36] Marazziti D. Mandillo S. Di Pietro C. Golini E. Matteoni R. Tocchini-Valentini G. P. (2007). GPR37 associates with the dopamine transporter to modulate dopamine uptake, and behavioral responses to dopaminergic drugs. *Proc. Natl. Acad. Sci. U.S.A.* 104 9846–9851. 10.1073/pnas.0703368104 17519329 PMC1887553

[B37] Meyer R. C. Giddens M. M. Coleman B. M. Hall R. A. (2014). The protective role of prosaposin, and its receptors in the nervous system. *Brain Res.* 1585 1–12. 10.1016/j.brainres.2014.08.022 25130661 PMC4529117

[B38] Meyer R. C. Giddens M. M. Schaefer S. A. Hall R. A. (2013). GPR37, and GPR37L1 are receptors for the neuroprotective, and glioprotective factors prosaptide, and prosaposin. *Proc. Natl. Acad. Sci. U.S.A.* 110 9529–9534. 10.1073/pnas.1219004110 23690594 PMC3677493

[B39] Morato X. Lujan R. Lopez-Cano M. Gandia J. Stagljar I. Watanabe M. (2017). The Parkinson’s disease-associated GPR37 receptor interacts with striatal adenosine A2A receptor controlling its cell surface expression, and function in vivo. *Sci. Rep.* 7:9452. 10.1038/s41598-017-10147-x 28842709 PMC5573386

[B40] Muratori F. Tonacci A. Billeci L. Catalucci T. Igliozzi R. Calderoni S. (2017). Olfactory processing in male children with autism: atypical odor threshold, and identification. *J. Autism Dev. Disord.* 47 3243–3251. 10.1007/s10803-017-3250-x 28744761

[B41] Nash H. H. Borke R. C. Anders J. J. (2001). New method of purification for establishing primary cultures of ensheathing cells from the adult olfactory bulb. *Glia* 34 81–87. 10.1002/glia.1043 11307157

[B42] Raucci F. Tiong J. D. Wray S. (2013). P75 nerve growth factor receptors modulate development of GnRH neurons, and olfactory ensheating cells. *Front. Neurosci.* 7:262. 10.3389/fnins.2013.00262 24409113 PMC3873506

[B43] Sbacchi S. Acquadro F. Calo I. Cali F. Romano V. (2010). Functional annotation of genes overlapping copy number variants in autistic patients: focus on axon pathfinding. *Curr. Genom.* 11 136–145. 10.2174/138920210790886880 20885821 PMC2874223

[B44] Sharifi N. Reuss A. E. Wray S. (2002). Prenatal LHRH neurons in nasal explant cultures express estrogen receptor beta transcript. *Endocrinology* 143 2503–2507. 10.1210/en.143.7.2503 12072381

[B45] Shi S. R. Chaiwun B. Young L. Cote R. J. Taylor C. R. (1993). Antigen retrieval technique utilizing citrate buffer or urea solution for immunohistochemical demonstration of, androgen receptor in formalin-fixed paraffin sections. *J. Histochem. Cytochem.* 41 1599–1604. 10.1177/41.11.7691930 7691930

[B46] Smith B. M. Giddens M. M. Neil J. Owino S. Nguyen T. T. Duong D. (2017). Mice lacking Gpr37 exhibit decreased expression of the myelin-associated glycoprotein MAG, and increased susceptibility to demyelination. *Neuroscience* 358 49–57. 10.1016/j.neuroscience.2017.06.006 28642167 PMC5562287

[B47] Smith N. J. (2015). Drug discovery opportunities at the endothelin B receptor-related orphan G protein-coupled receptors, GPR37, and GPR37L1. *Front. Pharmacol.* 6:275. 10.3389/fphar.2015.00275 26635605 PMC4648071

[B48] Sunkin S. M. Ng L. Lau C. Dolbeare T. Gilbert T. L. Thompson C. L. (2013). Allen Brain Atlas: an integrated spatio-temporal portal for exploring the central nervous system. *Nucleic Acids Res.* 41 D996–D1008. 10.1093/nar/gks1042 23193282 PMC3531093

[B49] Sykiotis G. P. Hoang X.-H. Avbelj M. Hayes F. J. Thambundit A. Dwyer A. (2010). Congenital idiopathic hypogonadotropic hypogonadism: evidence of defects in the hypothalamus, pituitary, and testes. *J. Clin. Endocrinol. Metab.* 95 3019–3027. 10.1210/jc.2009-2582 20382682 PMC2902061

[B50] Takahashi R. Imai Y. (2003). Pael receptor, endoplasmic reticulum stress, and Parkinson’s disease. *J. Neurol.* 250(Suppl. 3), III25–III29. 14579121 10.1007/s00415-003-1305-8

[B51] Tonacci A. Billeci L. Tartarisco G. Ruta L. Muratori F. Pioggia G. (2017). Olfaction in autism spectrum disorders: a systematic review. *Child Neuropsychol.* 23 1–25. 26340690 10.1080/09297049.2015.1081678

[B52] Topaloglu A. K. (2017). Update on the Genetics of Idiopathic Hypogonadotropic Hypogonadism. *J. Clin. Res. Pediatr. Endocrinol.* 9(Suppl. 2), 113–122. 10.4274/jcrpe.2017.S010 29280744 PMC5790323

[B53] Wray S. (2010). From nose to brain: development of gonadotrophin-releasing hormone-1 neurones. *J. Neuroendocrinol.* 22 743–753. 10.1111/j.1365-2826.2010.02034.x 20646175 PMC2919238

[B54] Wray S. Gahwiler B. H. Gainer H. (1988). Slice cultures of LHRH neurons in the presence, and absence of brainstem, and pituitary. *Peptides* 9 1151–1175. 10.1016/0196-9781(88)90103-9 3072535

[B55] Yang H. J. Vainshtein A. Maik-Rachline G. Peles E. (2016). G protein-coupled receptor 37 is a negative regulator of oligodendrocyte differentiation, and myelination. *Nat. Commun.* 7:10884. 10.1038/ncomms10884 26961174 PMC4792952

[B56] Zaghetto A. A. Paina S. Mantero S. Platonova N. Peretto P. Bovetti S. (2007). Activation of the Wnt-beta catenin pathway in a cell population on the surface of the forebrain is essential for the establishment of olfactory axon connections. *J. Neurosci.* 27 9757–9768. 10.1523/jneurosci.0763-07.2007 17804636 PMC1986640

