# Supplementary figures and images for: GPR37 Signaling Modulates Migration of Olfactory Ensheathing Cells and Gonadotropin Releasing Hormone Cells in Mice
Source: Front Cell Neurosci. 2019 May 9;13:200. doi: 10.3389/fncel.2019.00200 (PMC6521704; doi:10.3389/fncel.2019.00200)

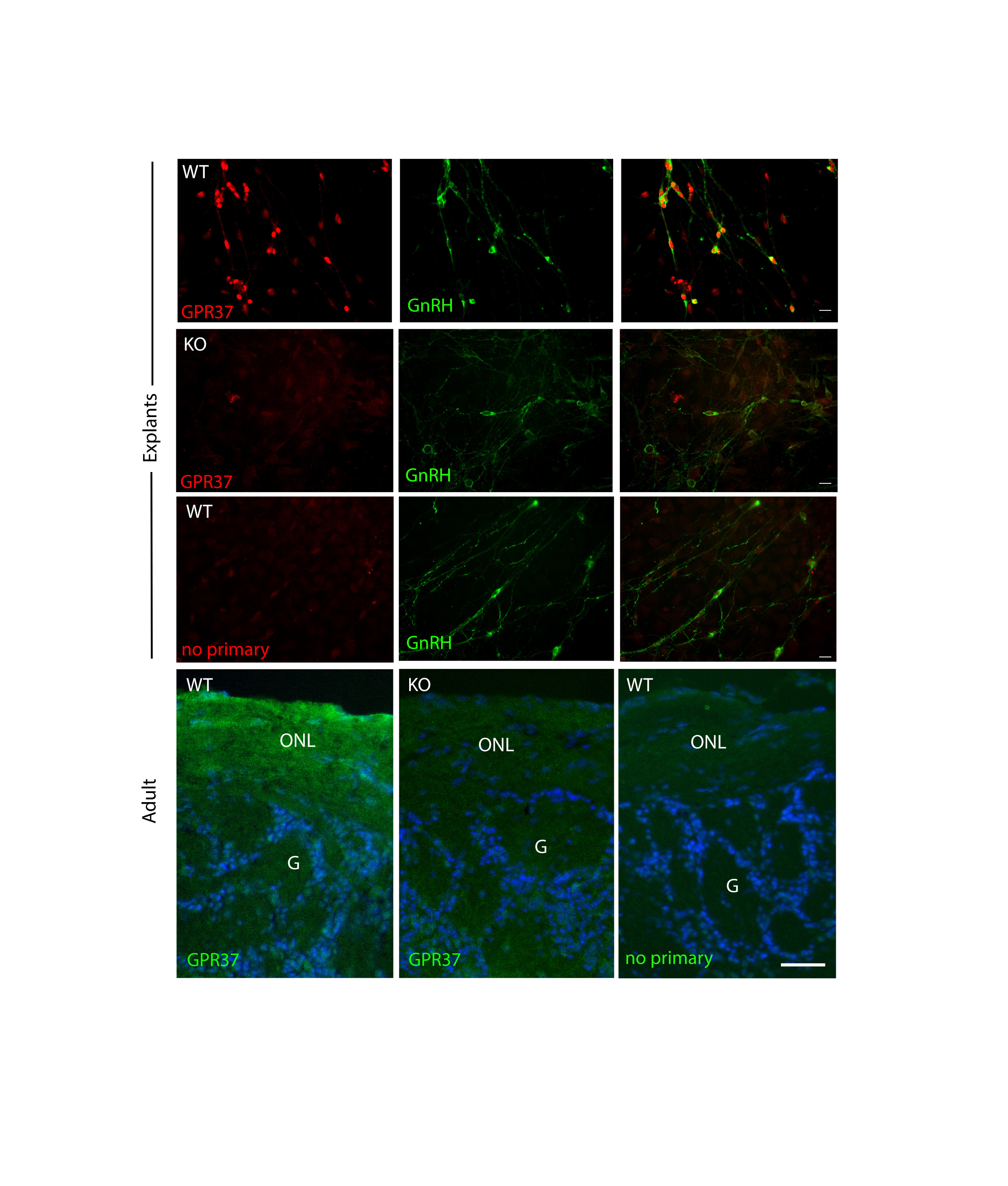

Supplement: FIGURE S1 — Specificity of GPR37 antibody. Double-label immunofluorescence on explants generated from WT and KO E11.5 embryos, maintained for 4 days in culture, fixed and stained for GPR37 (red, rabbit antibody) and GnRH (green, mouse antibody). A no primary control was run at the same time. Neither the KO nor the no primary control showed a red GPR37 signal. Single label immunofluorescence for GRP37 (green) was run on adult olfactory bulb sections from WT and KO littermates. A no primary control was run at the same time. GPR37 is only seen in the WT OB section, particularly in the olfactory nerve layer (ONL). G, glomeruli. Scale: Explant panels = 10 μm, adult panel: 50 μm. [file Image_1.JPEG]

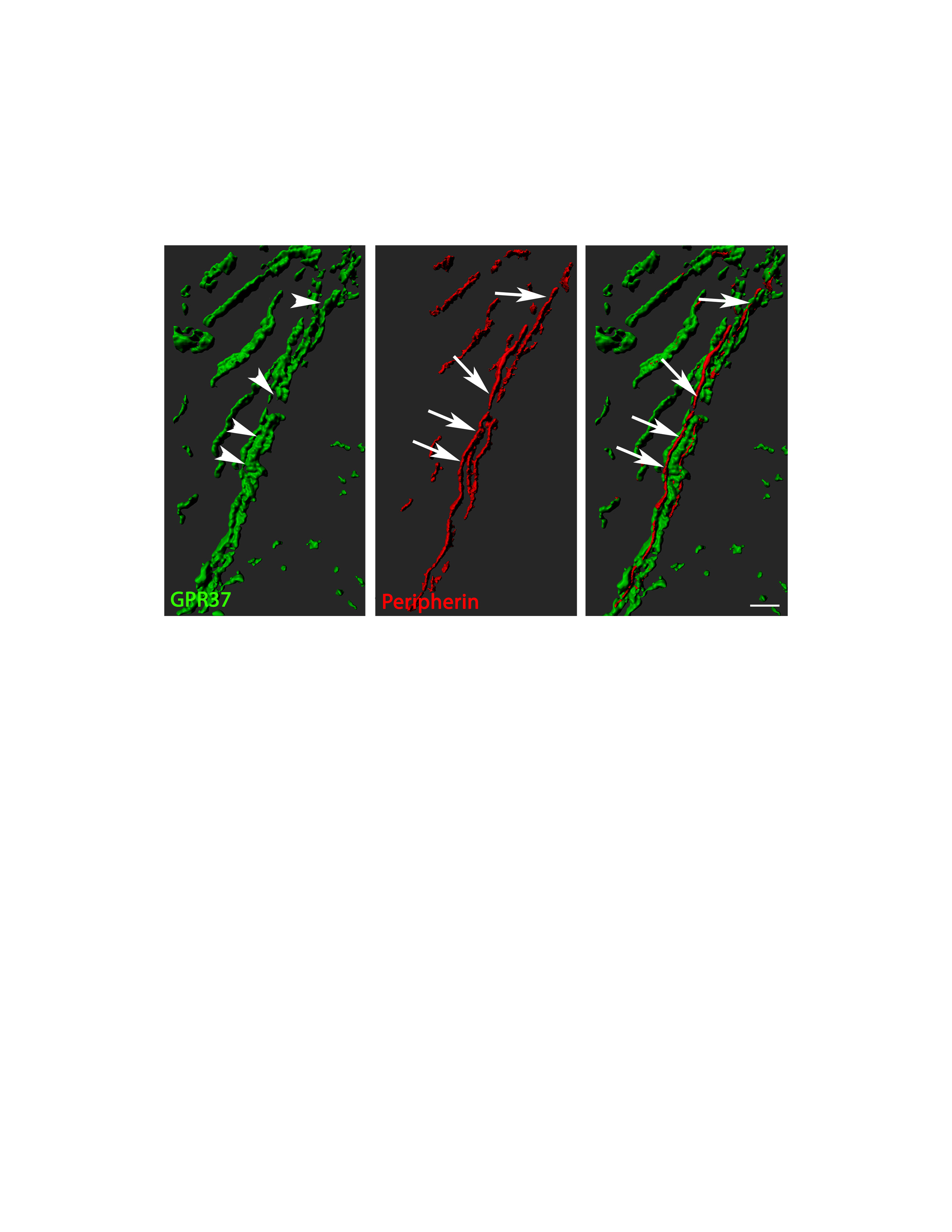

Supplement: FIGURE S2 — GPR37 is not expressed by olfactory axons in vivo. Imaris generated surface rendering processed from confocal stack, one of which is shown in Figure 2 E11.5. Reconstruction shows GPR37 negative “channels” (arrowheads) in GPR37 positive tracks (green). These “channels” contained peripherin positive fibers (red, arrows). Scale: 30 μm. [file Image_2.JPEG]

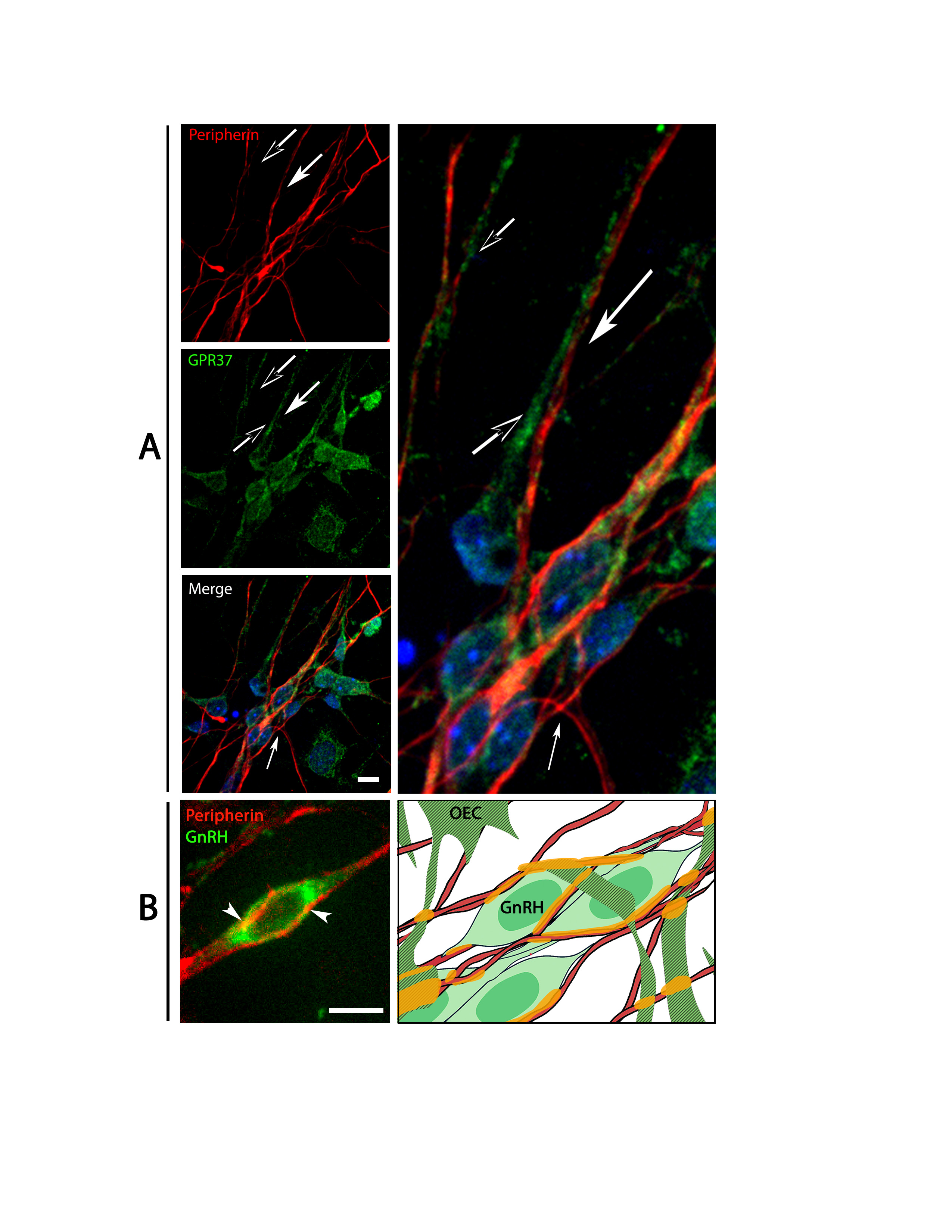

Supplement: FIGURE S3 — GPR37 is not expressed by olfactory axons in vitro. (A) 4DIV explant stained for peripherin (red, mouse antibody), which marks olfactory axons and GPR37 (green, rabbit antibody). In vitro, as in vivo, migratory tracks are composed of dense fibers and cells (see B right panel). However, peripherin fibers (white arrow) and GPR37 fibers (black arrows) are not double-labeled. False positive can occur as shown in (B). Left panel shows image of peripherin fiber (green) coursing over a GnRH cell (red). GnRH cells are not peripherin positive, yet yellow puncta are seen (arrowheads). Right panel shows schematic of the relationship between OECs which are GRP37 positive (green) and GnRH cells which are GPR37 positive (green) and peripherin fibers (red) which are GPR37 negative. As elements sit on each other false positives (orange) can occur. Scale: 10 μm. [file Image_3.JPEG]
